# West meets east: open up a dialogue on phytomedicine

**DOI:** 10.1186/s13020-021-00467-6

**Published:** 2021-07-19

**Authors:** Xiuzhu Li, Weijie Chen, Jesus Simal-Gandara, Milen I. Georgiev, Hongyi Li, Hao Hu, Xu Wu, Thomas Efferth, Shengpeng Wang

**Affiliations:** 1 Institute of Chinese Medical Sciences, State Key Laboratory of Quality Research in Chinese Medicine, University of Macau, Macao SAR Taipa, China; 2 Nutrition and Bromatology Group, Department of Analytical Chemistry and Food Science, Faculty of Food Science and Technology, University of Vigo-Ourense Campus, 32004 Ourense, Spain; 3 Laboratory of Metabolomics, The Stephan Angeloff Institute of Microbiology, Bulgarian Academy of Sciences, Plovdiv, Bulgaria; 4 Laboratory of Molecular Pharmacology, Department of Pharmacology, School of Pharmacy, Southwest Medical University, Luzhou, Sichuan China; 5 Institute of Pharmaceutical and Biomedical Sciences, Department of Pharmaceutical Biology, Johannes Gutenberg University, Mainz, Germany

**Keywords:** Phytomedicine, Chinese medicine, *Rhodiola*, Seabuckthorn, Fenugreek

## Abstract

The desire to extend the wisdom of traditional health systems has motivated the trade of many phytomedicine on a global scale for centuries, especially some dietary herbs, making a great overlap exits between western and eastern phytomedicine. Despite the communication since ancient times, a key disconnect still exists in the dialog among western and eastern herbal researchers. There is very little systematic effort to tap into the friction and fusion of eastern and western wisdom in utilizing phytomedicine. In this review, we analyzed the similarities and differences of three representative phytomedicine, namely *Rhodiola*, seabuckthorn, and fenugreek, aiming to open up new horizons in developing novel health products by integrating the wisdom of the east and the west.

## Background

The development of health products of phytomedicine has often stemmed from traditional or historical use, or from long-term evidence that consumption of phytomedicine is associated with better health outcomes [1]. Phytomedicine represent a collection of therapeutic knowledge that deeply rooted in a culture and formed the basis of early version of pharmacopoeias, which was based in large part on natural products originated from botanicals, animals, fungi, and minerals. As one of the most comprehensive and experienced form of ethnomedicine, the history of Chinese medicine can be traced back to at least 2000 years ago. The varied geographical features of China have endowed Chinese medicine with over 10 thousand kinds of phytomedicine. The Europe and America has also shared a very vibrant history of folk medicine, enriched with the various documented uses of phytomedicine [2].

The phytomedicine systems are always highly adaptive in nature and are open to adopt new imported species. Therefore, the desire to extend the wisdom of traditional health systems has motivated the trade of many phytomedicine on a global scale for centuries, especially some dietary herbs, making a great overlap exits between western phytomedicine and eastern phytomedicine. In the European Pharmacopeia (Version 9.5), a total of 219 phytomedicine aggregates are listed, of which 97 are native to Europe (44%) and 81 are from Asia (37%) [3]. For example, astragalus (*Huangqi*) is native to China since the *Shennong*’*s Herbal Classic* (200–245 CE) and gained its popularity in U.S. in the 1980's, while American ginseng (*Panax quinquefolium* L.) is native to eastern North America and then has been introduced to China with wide cultivation since eighteenth century.

As ethnologic phytomedicine are generated based on local cultures, resources, folkloric understandings and practices [4], western phytomedicine and eastern phytomedicine distinct at serval aspects (Table 1): (i) treating similar diseases by using different species belonging to the same genus (e.g. *Rholiola rosea* L. in Europe and *Rholiola crenulata* (Hook. f. & Thomson) H. Ohba in China); (ii) managing different diseases by using a same phytomedicine (e.g. sea buckthorn, turmeric and fenugreek); (iii) using different parts of a same phytomedicine for medicinal use (e.g. ginkgo leaf in Europe and ginkgo seeds in China). Despite the communication since ancient times, a key disconnect still exists in the dialog among western and eastern herbal researchers. There is very little systematic effort to tap into the friction and fusion of eastern and western wisdom in utilizing phytomedicine. In this study, several representative phytomedicine, namely *Rhodiola*, sea buckthorn and fenugreek, are selected for their long-term history of medical use, wide distribution, diverse clinical application and increasing global popularity. Following the analysis of the similarities and differences of the representative phytomedicine, we also discussed the future development of phytomedicine, aiming to open up new horizons in integrating the wisdom of the east and the west.

**Table 1:** Application of representative phytomedicine in the east and the west. Data source: WHO monograph on selective medicinal plants, Chinese Pharmacopeia, EU herbal monograph, ESCOP monograph and Commission E monograph

Common name	Species and medicinal applications in WHO monographs on selective medicinal plants	Species and medicinal applications in Chinese Pharmacopeia	Species and medicinal applications in EU herbal monograph	Species and medicinal applications in ESCOP monograph	Species and medicinal applications in Commission E monograph
*Rhodiola*	Not included	*Species: R. crenulata* *Medicinal parts:* dried root and rhizome *Medicinal use:* qi deficiency, blood stasis, chest *bi* disorder, heart pain, hemiplegia caused by wind-stroke, fatigue and panting	*Species: R. rosea* *Medicinal parts:* dried root and rhizome *Medicinal use: *relieve temporary symptoms of stress such as sensation of weakness and fatigue	Not included	Not included
Raspberry	Not included	*Species: Rubus chingii* Hu *Medicinal part:* dried fruit *Medicinal use:* seminal emission, spermatorrhea, enuresis, frequent urination, nourish the liver and improve vision	*Species: Rubus idaeus* L. *Medicinal part:* dried leaf *Medicinal use:* symptomatic relief of minor spasm associated with menstrual periods, symptomatic treatment of mild inflammation in the mouth or throat, and mild diarrhoea	Not included	Not included
Plantain	*Species: Plantago afra* L., *P. indica* L., *P. ovata* Forsk., or *P. asiatica *L. *Medicinal part:* dried, ripe seed *Medicinal use supported by clinical data:* treatment of chronic constipation, temporary constipation due to illness or pregnancy, irritable bowel syndrome, constipation related to duodenal ulcer or diverticulitis, softening the stools of those with haemorrhoids, or after anorectal surgery *Medicinal use described in pharmacopoeias and in traditional systems of medicine:* treatment of constipation, short-term symptomatic treatment of diarrhoea of various etiologies *Medicinal use described in folk medicine, not supported by experimental or clinical data:* treatment of rheumatic and gouty afflictions, glandular swelling, and bronchitis	*Species: P. asiatica* L. or *P. depressa* Willd *Medicinal part:* whole dried herb *Medicinal use:* heat strangury with chronic pain, edema, small quantity of urination, and diarrhea caused by summerheat-dampness, phlegm-heat cough, hematemesis, epistaxis, swelling abscess, sore and skin infections	*Species: P. lanceolata* L. *Medicinal part:* whole or fragmented, dried leaf *Medicinal use:* a demulcent for the symptomatic treatment of oral or pharyngeal irritations and associated dry cough	*Species: P. lanceolata* L. *Medicinal part:* whole or fragmented dried leaf and scape, or dried flowering aerial part *Medicinal use:* catarrh of the respiratory tract, temporary, mild inflammatory conditions of the oral and pharyngeal mucosa	*Species: P. lanceolata* L. and *P. major* L. *Medicinal part:* fresh or dried aboveground part *Internal medicinal use:* catarrhs of the respiratory tract and inflammatory alterations of the oral and pharyngeal mucosa *External medicinal use*: inflammatory reactions of the skin
Motherwort	Not included	*Species: Leonurus ja ponicus* Houtt *Medicinal part:* fresh or dried aerial part *Medicinal use:* menstrual irregularities, dysmenorrhea, amenorrhea, persistent flow of the lochia, edema, small quantity of urination, sore, ulcer, swelling, and toxin	*Species: L. cardiaca* L. *Medicinal parts:* whole or cut, dried flowering part *Medicinal use:* relieve symptoms of nervous tension; relieve symptoms of nervous cardiac complaints such as palpitations, after serious conditions have been excluded by a medical doctor	*Species: L. cardiaca* L. *Medicinal parts:* whole or cut flowering aerial part *Medicinal use:* mild cardiac complaints of nervous origin	*Species: L. cardiaca* L. *Medicinal part:* aboveground part *Medicinal use:* nervous cardiac disorders and as an adjuvant for thyroid hyperfunction
Fenugreek	*Species: Trigonella foenum-graecum* L. *Medicinal part:* dried ripe seed *Medicinal use supported by clinical data:* management of hypercholesterolaemia, and hyperglycaemia in cases of diabetes mellitus, prevention and treatment of mountain sickness *Medicinal use described in pharmacopoeias and in traditional systems of medicine: internal use for loss of appetite, and external use for local inflammations, treatment of pain, weakness and oedema of the legs* *Medicinal use described in folk medicine, not supported by experimental or clinical data: treatment of abdominal colic, bronchitis, diarrhoea, eczema, gout, indigestion, dropsy, fever, impotence, chronic cough*, *liver disorders, wounds and the common cold*	*Species: T. foenum-graecum* L. *Medicinal part:* seed *Medicinal use:* deficiency of kidney yang, deficiency cold in low origin, cold pain in low abdomen, abdominal pain caused by cold, abdominal colic, and cold-dampness tinea pedis	*Species: T. foenum-graecum* L. *Medicinal part:* seed *Medicinal use:* treat temporary lack of appetite and skin inflammations	Not included	*Species: T. foenum-graecum* L. *Medicinal part:* dried seed *Internal medicinal use:* loss of appetite and *External medicinal use*: a poultice for local inflammation
Turmeric	*Species: Curcuma longa* L. *Medicinal part:* dried rhizome *Medicinal use supported by clinical data:* treatment of acid, flatulent, or atonic dyspepsia *Medicinal use described in pharmacopoeias and in traditional systems of medicine:* treatment of peptic ulcers, and pain and inflammation due to rheumatoid arthritis and of amenorrhoea, dysmenorrhoea, diarrhoea, epilepsy, pain, and skin diseases *Medicinal use described in folk medicine, not supported by experimental or clinical data:* treatment of asthma, boils, bruises, coughs, dizziness, epilepsy, haemorrhages, insect bites, jaundice, ringworm, urinary calculi, and slow lactation		*Species: C. longa* L. *Medicinal part:* whole, cured (by boiling or steaming), dried rhizome *Medicinal use:* relieve digestive disturbances, such as feelings of fullness, slow digestion and flatulence	Not included	*Species: C. longa* L. *Medicinal part:* scalded and dried rhizome *Medicinal use:* dyspeptic conditions
St. John's Wort	*Species: Hypericum perforatum* L. *Medicinal part:* dried flowering tops or aerial parts *Medicinal use supported by clinical data:* symptomatic treatment of mild and moderate depressive episodes *Medicinal use described in pharmacopoeias and in traditional systems of medicine:* externally for the treatment of minor cuts, burns and skin ulcers, topically for viral infections *Medicinal use described in folk medicine, not supported by experimental or clinical data:* treatment of inflammation of the bronchi and urogenital tract, treatment of biliary disorders, bladder irritation, the common cold, diabetes mellitus, dyspepsia, haemorrhoids, neuralgia, migraine headaches, sciatica and ulcers, used as a diuretic, an emmenagogue and an antimalarial agent	*Species: H. perforatum* L. *Medicinal part:* dried aerial part *Medicinal use:* liver qi depression, moodiness, depression in the heart and chest, joint swelling, pain, acute mastitis, and oligogalactia	*Species: H. perforatum* L. *Medicinal part:* whole or cut flowering top *Traditional use:* relief of temporary mental exhaustion, minor inflammations of the skin (such as sunburn) and as an aid in healing of minor wounds, relief of mild gastrointestinal discomfort *Well-established use:* treatment of mild to moderate depressive episodes, short term treatment of symptoms in mild depressive disorders	*Species: H. perforatum* L. *Medicinal part:* whole or cut, dried flowering tops *Medicinal use*: mild to moderate depressive episodes	*Species: H. perforatum* L. *Medicinal part:* dried aboveground part *Medicinal use:* psychovegetative (psychoautonomic) disturbances, depressive moods, anxiety, and nervous unrest
Burdock	Not included	*Species: Arctium lappa* L. *Medicinal part:* dried fruit *Medicinal use:* common cold caused by wind-heat, cough, profuse sputum, measles, rubella, swelling and sore of throat, mumps, erysipelas, swelling abscess, and skin infections	*Species: A. lappa* L. *Medicinal part:* dried, whole or cut root *Medicinal use:* increase the amount of urine to achieve flushing of the urinary tract as an adjuvant in minor urinary tract complaints; temporary loss of appetite; treatment of seborrhoeic skin conditions	*Species: A. lappa* L. *Medicinal part:* dried, whole or cut root *Internal medicinal use:* seborrhoeic skin, eczema, furuncles, acne, psoriasis, an adjuvant in minor urinary tract complaints *External medicinal use*: seborrhoeic skin, eczema, furuncles, acne	Not included
Centella	*Species: Centella asiatica* (L.) Urban *Medicinal part:* dried aerial part *Medicinal use supported by clinical data:* treatment of wounds, burns, and ulcerous skin ailments, and prevention of keloid and hypertrophic scars *Medicinal use described in pharmacopoeias and in traditional systems of medicine:* treatment of leprous ulcers and venous disorders *Medicinal use described in folk medicine, not supported by experimental or clinical data:* therapy of albinism, anaemia, asthma, bronchitis, cellulite, cholera, measles, constipation, dermatitis, diarrhoea, dizziness, dysentery, dysmenorrhoea, dysuria, epistaxis, epilepsy, haematemesis, haemorrhoids, hepatitis, hypertension, jaundice, leukorrhoea, nephritis, nervous disorders, neuralgia, rheumatism, smallpox, syphilis, toothache, urethritis, and varices; and as an antipyretic, analgesic, anti-inflammatory, and "brain tonic" agent	*Species: C. asiatica* (L.) Urban *Medicinal part:* dried whole herb *Medicinal use*: ampness-heat jaundice, diarrhea caused by summer-heat, stone strangury, blood strangury, swelling abscess, sore and toxin, and traumatic injuries	*Species: C. asiatica* (L.) Urban *Medicinal part:* aerial part *Medicinal use:* wound healing and memory enhancement	Not included	Not included

## Rhodiola

*Rhodiola* genus, a worldwide phytomedicine belonging to the plant family Crassulaceae, has a long history of being used to treat diarrhea, headache, hernias and hysteria, to prevent high-altitude sickness, and to improve symptoms of depression as well as to enhance physical strength and endurance in both Europe and Asia [5, 6]. *Rhodiola* has also been a food crop since ancient times, and now it is being used as a food ingredient and developed as an additive in cosmetics [5, 7, 8]. Several lines of evidence have shown that *Rhodiola* possesses numerous activities including antioxidant, anti-aging, anti-tumor, anti-stress, anti-fatigue, anti-radiation, anti‐inflammation, immunomodulatory and blood-glucose-lowering [8–11].

The genus *Rhodiola* grows in cold mountainous areas such as rock ledges, precipices, tundra, brooks, and river banks in northern hemisphere including North and Central Europe, North America and Asia [12]. There are more than 100 species of *Rhodiola* in the world with similar tissue structure and medicinal material morphology and the original genus *Rhodiola* is thought to firstly appear in the mountainous areas in Southwest China and the Himalayas [8, 13]. Among the diverse of *Rhodiola*, *R. crenulata* and *R. rosea* are the most two well recognized and studied species, and between western countries and China, the different use of *Rhodiola* mainly shows on two species which are *R. crenulata* and *R. rosea*.

In China, *Rhodiola* has been used in traditional Tibetan medicine for over 1000 years and was recorded with folk use of treating pneumonia, cough, hemoptysis and abnormal leucorrhea in the Tibetan medicine books such as “Four Medical Code” (*rGyud-bzhi* in Tibetan, *Si Bu Yi Dian* in Chinese, 800 AD) [14] and “Jing Zhu Materia Medica” (*Shel Gong Shel Phreng* in Tibetan, *Jing Zhu Ben Cao* in Chinese, 1745–1840 AD) [15]. Nowadays, over 70 species of *Rhodiola* have been recorded in China. *R. crenulata*, Dahua Hongjingtian in Chinese, which is the only official specie documented in Chinese Pharmacopoeia. Its root and rhizome are widely used in Tibetan medicine and traditional Chinese medicine for its therapeutic effect in tonifying qi and activating blood, as well as in alleviating heart pain, hemiplegia caused by wind-stroke, fatigue and panting [4, 16]. *R. crenulata* has been found in western Sichuan, northern Yunnan and eastern Tibet in China and grows nearly exclusively in rock crevices on peaks of mountain with altitude from 4300 to 5600 m, making it one of the highest vascular plants on Qinghai-Tibetan Plateau. [13, 17].

*R. rosea*, also known as Roseroot, Golden Root, Arctic Root and Orpin Rose, is the most commonly used species in western countries [13, 18]. In Russia, Scandinavia and some other countries, *R. rosea* have been used as traditional medicine since several hundred years ago [19]. *R. rosea* was first recorded with its therapeutic use in the medical book *De Materia Medica* by the Greek physician Dioscorides in 77 AD [20]. In 1969, the Soviet Ministry of Health approved and registered the medicinal application of *R. rosea.* Since 1985, *R. rosea* has been recorded in Herbal Medicinal Product in Sweden. Nowadays, *R. rosea* is documented by European Medicines Agency (EMA) with medicinal use of relieving temporary symptoms of stress such as sensation of weakness and fatigue. (http://www.ema.europa.eu/ema). *R. rosea* grows on sea cliffs and in crevices of mountain rocks with an elevation which reach up to 2280 m and distribute in Europe (mainly in Arctic regions and Britain), North America and Asia (mainly in Siberia) [18, 21].

**Fig. 1:**
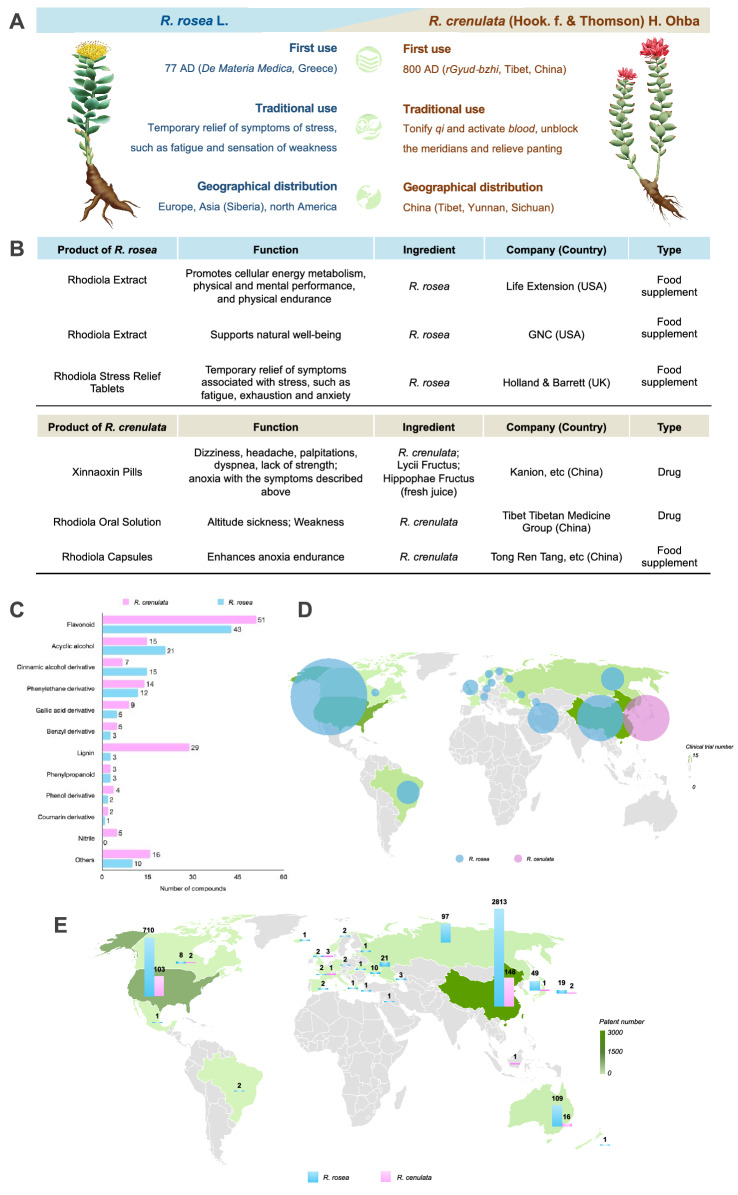
Comparison of history, products, active constituents, clinical trials and patens of two *Rhodiola* species. **A** Basic information of *R. crenulata* and *R. rosea*. **B** Representative products developed based on *R. crenulata* and *R. rosea*. **C** Number of main active constituents contained in *R. crenulata* and *R. rosea*. **D** Advanced in clinical trials of *R. crenulata* and *R. rosea*. Data collected from PubMed (searching term of “*Rhodiola*” plus filter of “Clinical trial”, language limited to English) and ClinicalTrials.gov (searching term of “*Rhodiola*”) as of 15 June 2021. E. Patent application of *R. crenulata* and *R. rosea.* Data collected from the Lens (www.lens.org, Searching term: *Rhodiola*) as of 15 June 2021

There has been an increasing demand for the *Rhodiola* products in recent years due to their multiple healthy benefits [22]. In China, the most widely traded species is *R. crenulata*. According to the data collected from China Food and Drug Administration (CFDA), in 2018, there were 126 domestic *Rhodiola* products in China, of which capsules account for the most (56.4%), followed by tablets and pills (19%), oral solution (7.9%), granules (5.6%), drinks (4.8%), teas (2.4%), granules (5.6%), drinks (4.8%) and alcohol (1.6%). Xinnaoxin Pills and *Rhodiola* Oral Solution are the approved drugs while *Rhodiola* Capsules are the approved health food (Fig. 1B). On the other hand, *R. rosea* is the most widely developed species for commercial trade [12]. As stated by Galambosi, more than 46 companies in the world sell the products of *R. rosea* while 30 companies supply the products as food ingredients [23]. Currently, herbal products containing *R. rosea* are mainly used as dietary supplements and are easily available from retail stores and on the internet in western countries [21, 24]. In Fig. 1B, three kinds of *R. rosea* products are showed and all of them are herbal supplements. However, like *R. crenulata*, the growing demand for *R. rosea* has also led to its shortage of resource [25].

However, it is the growing demand of *Rhodiola*-based products that has led to the scarcity of *Rhodiola* resource. Since the 1980s, the use of *R. crenulata* has increased without proper controlled in southwestern China and this has resulted in the deforestation. Moreover, *R. crenulata*, as well as other species of *Rhodiola*, have been considered for being included on the List of Wild Plants under State Priority Conservation to avoid species extinction [26]. For *R. rosea*, the intensive collection has also resulted in the scarce natural resource, making *R. rosea* one of the listed endangered plant species in some countries such as Britain, Russia, Bulgaria and Slovakia [12]. Apart from the species conservation, commercial cultivation is also being practiced for achieving the sustainable use of *R. crenulata* and *R. rosea* although the cost of cultivation is high due to the long cultivation period of approximately five years [27]. In western countries, such as Britain, Finland, Denmark, Switzerland, Sweden, Norway, Slovenia, Canada, Bulgaria, Russia and America, cultivation projects of *R. rosea* are being conducted [27]. While in China, cultivation trials of both *R. crenulata* and *R. rosea* have been implemented in Tibet. Cunningham et al. also pointed out that cultivation of *Rhodiola* in China can use successful experience of *R. rosea* cultivation from Finland, Bulgaria, Slovenia and Canada for reference.

*Rhodiola* genus could be differentiated from other plants by specific makers of its eight compositions which are salidroside, tyrosol, rosavin, rosarin, rosin, catechin, rhodionin and gallic acid [21]. Among these compositions, salidroside exists in all species of *Rhodiola* and R. crenulata has the highest content [28]. Salidroside is shown to have effect of modulating the cellular energy status in diverse cell lines by activating the AMPK pathway. Various activities of *Rhodiola* such as antioxidant, anti-fatigue, anti-stress, and anti-inflammatory are ascribed to salidroside [29]. While rosavins (rosavin, rosarin, rosin), which are demonstrated to have a therapeutic function of anti-depression, are the specific components of *R. rosea* [30, 31]. Another composition that contributes to distinguishing these two species is 0.05% essential oils contained in *R. rosea,* which result in rose-like and stronger scent of *R. rosea* compared with scent of *R. crenulata* [7, 19]. According to a comprehensive review by Tao et al. [16], 160 compounds of *R. crenulata* have been reported and more than 100 compounds have been found from *R. rosea*. As shown in Fig. 1C, *R. crenulata* and R. rosea share similar feature in constituent subgroups including flavonoids and their glycosides, acyclic alcohol derivatives, cinnamic alcohol derivative, phenylethane derivatives, gallic acid and its derivatives, benzyl and phenol derivatives, lignin, phenylpropanoid derivatives, nitrile derivatives, and other compounds, except that the number of lignin compounds from *R. crenulata* (29 lignins reported) is higher than that of *R. rosea* (3 lignins reported). In addition, since salidroside is believed to be the main active constituent of *Rhodiola* species and rosavins are the specific marker of *R. rosea*, the quality control standard of *R. rosea* extracts is usually set to contain a minimum of 3% rosavins and 0.8–1% salidroside as the ratio of these two compounds, which exist in wild *R. rosea* roots is about 3: 1 [12, 32]. While for *R. crenulata* in Chinese Pharmacopoeia (version 2020), it is stipulated that at least 0.5% of salidroside in its dry root and rhizome should be contained [33].

Previous studies have been conducted to seek the differences between *R. crenulata* and *R. rosea* in terms of their pharmacological activities. Abidov et al. studied the effect of oral treatment with extracts from *R. rosea* (50 mg/kg) and *R. crenulata* (50 mg/kg) roots on the duration of exhaustive swimming and ATP content in mitochondria of skeletal muscles in rats. The results showed that treatment with *R. rosea* prolonged the length of exhaustive swimming significantly by 24.6% compared with the control group and the group with *R. crenulata* treatment. *R. rosea* extract could also activate the synthesis or resynthesis of ATP in mitochondria and stimulate reparative energy processes after intense exercise. [34]. Furthermore, a study compared the effectiveness of *R. crenulata* and *R. rosea* on management of Type II diabetes and hypertension [35]. Inhibiting α-amylase and α-glucosidase is an important management of Type II diabetes. [36] However, excessive inhibition of α-amylase in pancreas may result in the side-effect such as flatulence or even diarrhea. Therefore, lower inhibition of α-amylase combined with higher inhibition of α-glucosidase has been suggested to be an effective strategy for the treatment of Type II diabetes. In addition, hypertension, which is a long-term complication of diabetes and also a risk factor for cardiovascular disease (CVD), can be well managed through the proper inhibition of angiotensin I-converting enzyme (ACE). Kwon Y et al. investigated on the inhibitory activity of α-amylase, α-glucosidase and angiotensin converting enzyme (ACE) of *R. rose* and *R. crenulata*. Results indicated that ethanol extracts of *R. crenulata* (IC_50_, 120.9 μg total phenolic/mL) showed higher inhibitory activity against α-amylase than ethanol extracts of *R. rosea* (IC_50_, 173.4 μg total phenolic/mL), while ethanol extract of *R. rose* (IC_50_, 44.7 μg total phenolic/mL) showed better activity in inhibiting α-glucosidase than ethanol extract of *R. crenulata* (IC_50_, 60.2 μg total phenolic/mL). Moreover, ethanol extracts of *R. rose* also had higher activity in inhibiting rabbit lung ACE (38.5%) than ethanol extract of *R. crenulata* (11.2%) [35].

To find out the similarities and differences in status of clinical trials between *R. crenulata* and *R. rosea* in different regions, information of clinical trials of these two species were collected and analyzed (Fig. 1D). It is obviously that there are more clinical trials of *R. rosea* than that of *R. crenulata* from a global perspective. Also, clinical trials of *R. rosea* have been conducted widely in the world, mainly clustering in Europe and the U.S. In contrast, clinical trials of *R. crenulata* have been conducted in China only. In China, clinical trials of *R. rose* have been conducted as well but their number is not as many as that of *R. crenulata*. Compared with *R. rosea, R. crenulata* is the main research direction in China.

In regard to patent application, as shown in Fig. 1E, there have been 3,859 *R. rosea*-based patents, which are approximately 14 times higher than that of *R. crenulata* globally. In addition, countries/regions that have *R. rosea*-based patents are almost 19 times as many as those have *R. crenulata*-based patents. In other words, compared with *R. crenulata*-based patents*, R. rosea*-based patents have a wider distribution in countries/regions. Besides, for both *R. crenulata*-based patents and *R. rosea*-based patents, China has the largest amount, followed by the U.S.

As a traditionally considered safe phytomedicine, the low risk of toxicity of *Rhodiola* has been supported by animal studies with results of no or little toxicity, either acute or chronic [37–40]. In several clinical trials of *R. rosea* and *R. crenulata*, although some adverse events such as loss of appetite, headache, diarrhea, throat sore and xerostomia have been reported, all of them were mild [41–43]. However, combinational use of *Rhodiola* with conventional drugs was reported to lead to herb‐drug interactions, which may increase unpredictable risks and needs further exploration [16].

## Sea buckthorn

**Fig. 2:**
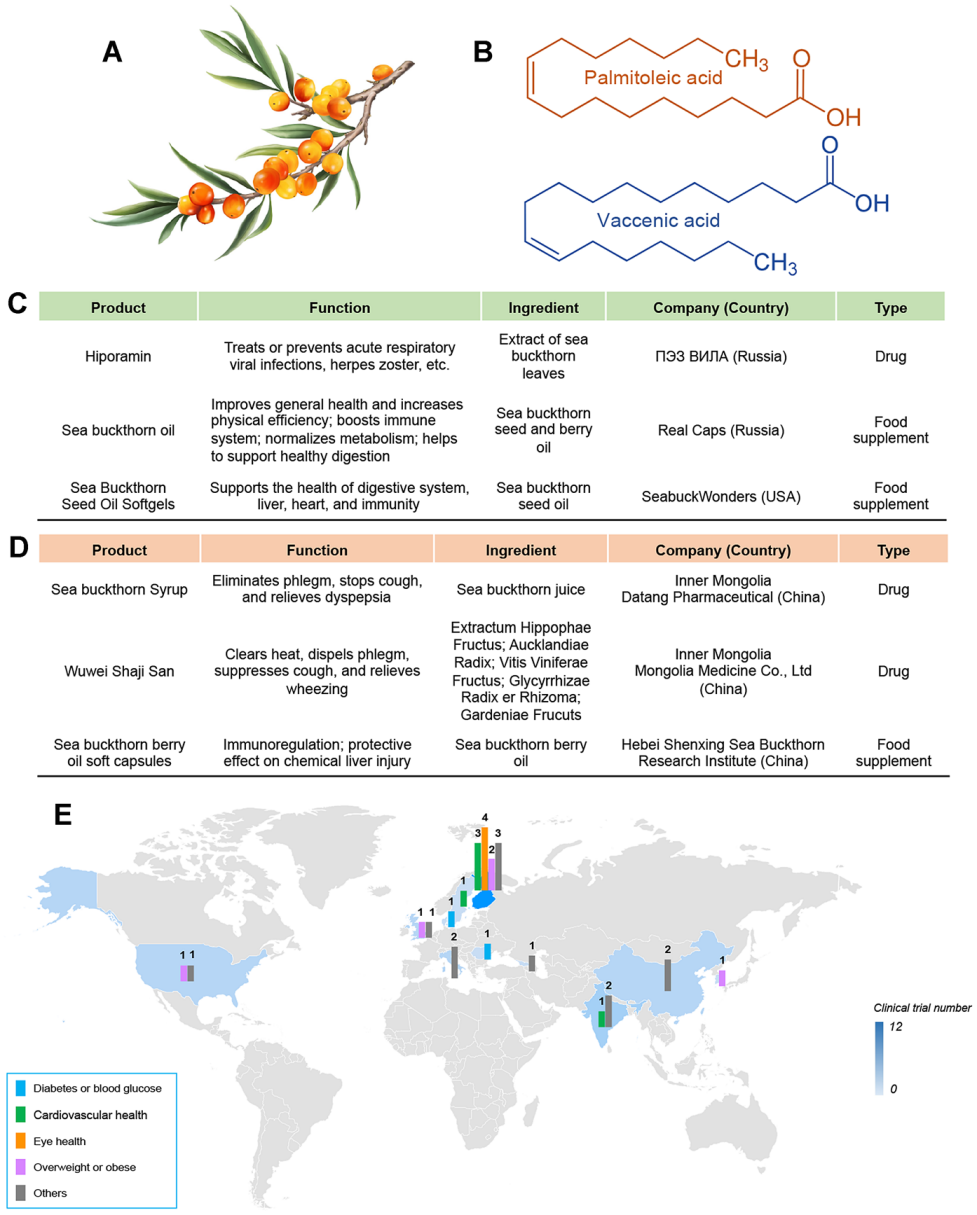
Plant morphology, representative products, and clinical studies of sea buckthorn. **A** Plant morphology of *Hippophae rhamnoides* L. (Sea buckthorn). **B** Chemical structure of two most common omega-7 fatty acids in sea buckthorn, namely palmitoleic acid and vaccenic acid. **C** Representative products developed based on sea buckthorn from western countries. **D** Representative products developed based on sea buckthorn from eastern countries. **E** A summarize of clinical trials of Sea buckthorn. Data collected from PubMed (searching term of “*Hippophae rhamnoides*” or “sea buckthorn” plus filter of “Clinical trial”, language limited to English) and ClinicalTrials.com (searching term of “*Hippophae rhamnoides*” or “sea buckthorn”) as of 15 June 2021

*Hippophae rhamnoides* L., commonly known as sea buckthorn (Fig. 2A), and native to Asia and Europe, is a hardy deciduous shrub belonging to the plant family Elaeagnaceae and can be further divided into eight subspecies. It mainly grows in river valley or on seacoast with gravel and sandy soil [44–46] and has a long history of being used as food, fodder and folk medicine in European and Asian countries. The earliest record of its medicinal use can track back to 800 A.D. in the Tibetan medicine books “Four Medical Code” (rGyud-bzhi in Tibetan, Si Bu Yi Dian in Chinese) [47]. Nowadays, in addition to the edible and medical values, sea buckthorn is also used for afforestation in desert due to its outstanding abilities to resist wind and conserve water and soil. Considering its multiple values, sea buckthorn has been planted in various regions and is now widely distributed in Central and Northern Europe, Central Asia, Russia, China, Mongolia, Canada and America [45, 48]. Different organs of sea buckthorn can be utilized, especially its berries which are demonstrated to be of high nutritional value [49].

In general, the sea buckthorn berries contain abundant vitamins (notably C and E) as well as other antioxidants such as fatty acids, flavonoids, phenolics, organic acids and carotenoids [50]. Compared with other fruits, sea buckthorn berries are the unique one on account of their rich content of omega-7 group (Fig. 2B), which are higher than that in any other ones. Besides, sea buckthorn berries are called “king of vitamin C” as the vitamin C content is claimed to be among the richest content in all the fruits and vegetables [48, 51, 52]. These bioactive compounds that the sea buckthorn contains also contribute to its medicinal properties. Traditionally, sea buckthorn is mostly used in relieving cough, treating conditions of digestive system as well as some skin problems [53, 54]. Sea buckthorn is a well-tolerated phytomedicine with no or few side effects, which is supported from acute and subchronic toxicity studies in animals [55, 56]. Several clinical studies have also demonstrated that there were no adverse events after the administration of sea buckthorn [57–60].

As a globally popular phytomedicine, the traditional use of sea buckthorn has been different between western and eastern countries since ancient time. In ancient Greece, local people used the leaves and twigs of sea buckthorn to feed animals for gaining weight and shining coat, especially for horses. Interestingly, the Latin name “Hippophae”, meaning shining horse, just derived from this traditional use [61]. In Russia, sea buckthorn berries are mostly used to treat gastrointestinal disorders and skin conditions such as psoriasis, eczema, lupus, frostbite and burns. Besides, Russian also used sea buckthorn to prevent rheumatism, eliminate internal blood clots as well as to treat jaundice, hepatitis, asthma [61–63]. While in China, the sea buckthorn berries have long been used for relieving cough and sputum, aiding digestion, activating blood and dissolving stasis in both traditional Chinese medicine and Tibetan medicine. Moreover, in Tibetan medicine, more conditions including colds, fever, inflammation, toxicity, abscesses, constipation, pulmonary disorders and gynecological diseases have been treated with sea buckthorn involved [49, 61]. As for other Asian countries, people in Mongolia use the berries to treat the same conditions as in Chinese medicine. Furthermore, they also used extracts of branches and leaves to treat colitis and enterocolitis for human and animals. While for countries in Central Asia, the berries and the leaves can be used to treat gastrointestinal disorders and skin conditions. Moreover, the berries can also be used to treat hypertension while the leaves can also be used to treat rheumatoid arthritis. Particularly, in Tajikistan, the flowers are used as skin softener [49, 61, 63].

Since 1977, the dry berries of sea buckthorn have been documented in Chinese Pharmacopoeia with therapeutic use of relieving cough and sputum, aiding digestion, activating blood and dissolving stasis which was based on the traditional use in Chinese medicine [33, 64]. While in Russia, the time when sea buckthorn was listed as an official drug is approximately 20 years earlier, i.e. in 1950s, sea buckthorn oil was documented in Russian Pharmacopeia and the official medicine of former Soviet Union as an anti-inflammatory aid. Actually, since 1940s, the sea buckthorn industry in Russia has become vibrant as the researchers began to study the bioactive compounds in sea buckthorn. In China, the research of sea buckthorn was initiated several decades later (i.e., in 1980s) than in Russia although China is the first country where sea buckthorn was medically used as recorded [65]. During that period, most of the studies on sea buckthorn have been originated in Russia and China and the therapeutic use of sea buckthorn has shifted from traditional use to evidence-based clinical use gradually [66]. For example, since 1950s when Russian initiated the clinical studies of sea buckthorn, medicinal preparations of sea buckthorn have been clinically used to treat gastric ulcers, oral inflammation radiation damage and burns in both former Soviet Union and China [67]. It was only in recent years that more and more studies have been conducted worldwide due to the increasing interest towards sea buckthorn utilization. More evidence-based therapeutic properties of sea buckthorn have been known to people such as antioxidant, anticancer, anti-inflammatory, antiviral, antibacterial, antiatherogenic, immunomodulatory, hepatoprotective, lowing blood sugar, preventing gum bleeding, protecting and recuperating mucosa of stomach or other organs, etc. Besides, it also shows potential to protect the cardiovascular system [61, 62, 67–73].

Nowadays, various forms of sea buckthorn products are on the market including juice, oils, powder, candies, tea, jellies, pigment, food additives, shampoos, cream and cosmetics [45, 74]. In addition, food supplements and drugs are the two important product forms of sea buckthorn. In Fig. 2C, D, serval sea buckthorn products of food supplements and drugs are listed. Specially, in Russia, the drug Hiporamin, extract of sea buckthorn leave, is used to treat serval kinds of viral infections based on the known bioactivity of antiviral of sea buckthorn. While in China, the drugs based on sea buckthorn still focus on the traditional use of suppressing cough, dispelling sputum and promoting digestion.

Due to the increasing desire for utilizing sea buckthorn, clinical trials based on sea buckthorn have been conducted in order to provide more further evidence for the medicinal use of sea buckthorn. In the 1950s, Russia initiated the first clinical study of sea buckthorn [64, 65, 75]. As shown in Fig. 2E, the number of clinical trials in western countries are almost four times as many as those in eastern countries so far. These clinical trials have focused on the medicinal use in several conditions such as diabetes or blood glucose, eye health, overweight or obese as well as cardiovascular health. Among these conditions, cardiovascular health and overweight or obese are the most concerned conditions and clinical studies on them have been conducted in both western and eastern countries. Especially, in addition to the above conditions, scientists from Finland discovered the potential of sea buckthorn oil in attenuating the symptom of dry eye and the increase in tear film osmolarity [76]. Moreover, another clinical study, also in Finland, was conducted to try to explore the potential mechanism of effect on treating dry eye with sea buckthorn. Based on the results, the researchers claimed that the eicosanoids produced from the fatty acids in sea buckthorn oil or the tocopherols and carotenoids in sea buckthorn oil might positively affect the differentiation of the meibomian gland cells and might be responsible for improving the inflammation [77].

## Fenugreek

**Fig. 3:**
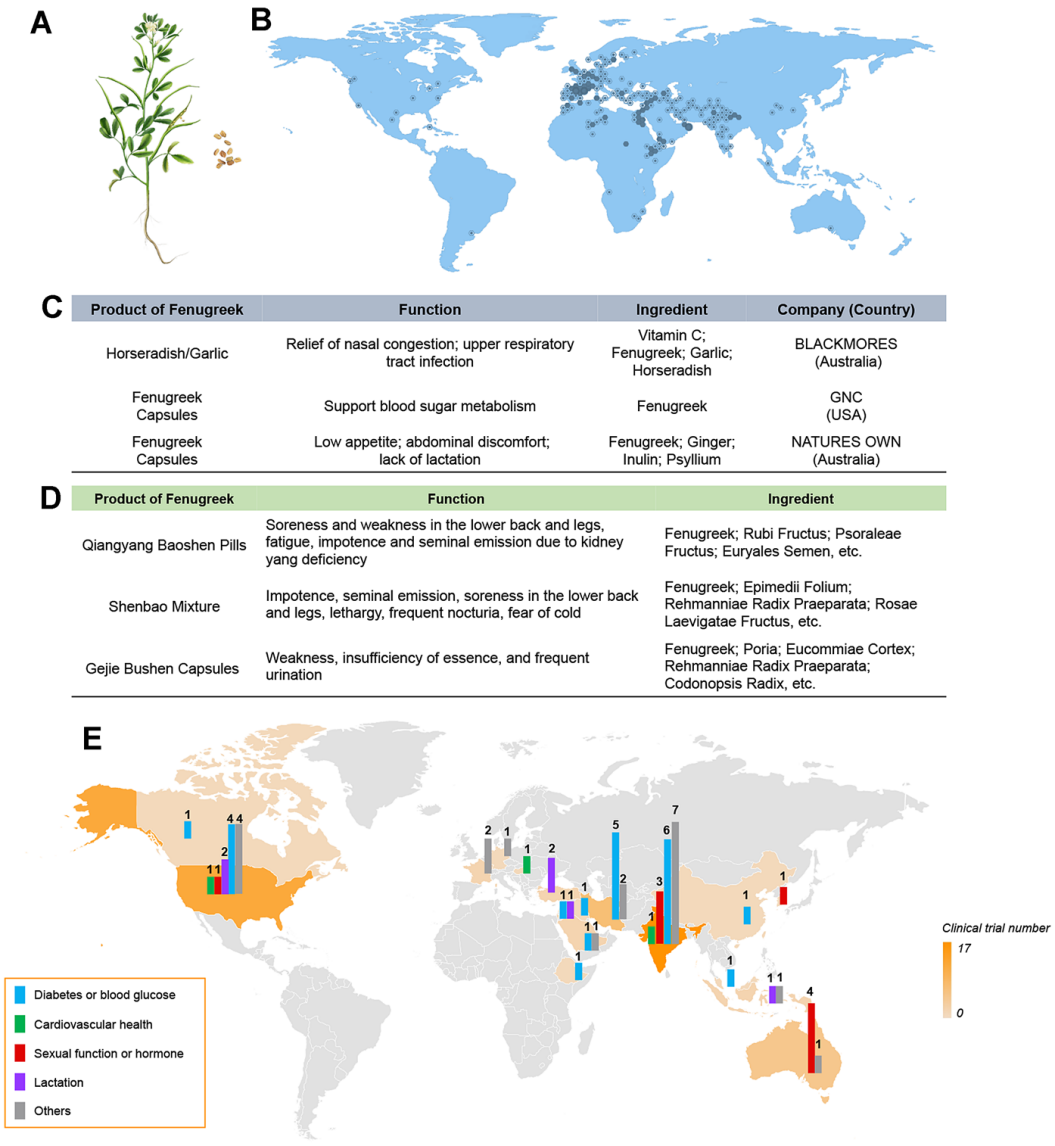
Plant morphology, geographical distribution, products, and clinical studies of fenugreek. **A** Plant morphology of *Trigonella foenum-graecum* L. (Fenugreek). **B** Geographical distribution of fenugreek. **C** Representative products developed based on fenugreek from western countries. **D** Representative products developed based on fenugreek from eastern countries. **E** Clinical trials of Sea buckthorn. Data collected from PubMed and ClinicalTrials.com as of June 2021 (Pubmed: searching term of “*Trigonella foenum-graecum*” or “fenugreek” plus filter of “Clinical trial”, language limited to English); ClinicalTrials.gov: searching term of “*Trigonella foenum-graecum*” or “fenugreek”)

Fenugreek (*Trigonella foenum-graecum* L.) (Fig. 3A) is one of the oldest phytomedicine native to western Asia and southeastern Europe (the Mediterranean region) and is now being introduced into over 50 countries across Asia, Africa, the Americas, and Europe [78] (Fig. 3B). Fenugreek contains a rich source of bioactive constituents, including polysaccharides, alkaloids, polyphenols and saponins [79], which multiple bioactivities has fueled the fast-growing demand of fenugreek in global pharmaceutical, nutraceutical, and functional food industries. Regarding the toxicity, only mild adverse events such as dyspepsia, diarrhea and abdominal distension were found in clinical application of fenugreek [80].

For centuries, fenugreek has been used in managing various human ailments, while the global introduction has spawned the diversified consumption patterns of fenugreek in different parts of the world. As a cattle fodder, fenugreek plants were used to treat sick animals in early Greek before its popularity in human wellbeing. Egyptian practitioners used fenugreek to treat skin wounds and combat fevers [81]. In India, fenugreek is commonly consumed as a food flavor and medicinally used in Ayurvedic medicine to treat indigestion and baldness, and to induce lactation [82]. In nineteenth century, fenugreek gained reputation in America as a treatment for female discomforts like dysmenorrhea and was listed as a key ingredient in Lydia Pinkham’s Vegetable Compound [83]. Fenugreek also have been adopted in Europe to treat temporary lack of appetite and skin inflammations (http://www.ema.europa.eu) [79]. Since travelled to China and first recorded in *Jiayou Medical Herbs* (1060 A.D.), fenugreek has been applied as a tonic Chinese medicine to warm and tonify kidney-yang, dissipate cold and relieve pain.

Nowadays, the potential benefits of fenugreek have come to light and some drugs and food supplements have been on the market. In these products, fenugreek is used alone or in combination with other tonic phytomedicine. As shown in Fig. 3C, for the two products from Australia, fenugreek is used in combination with two or three other phytomedicine. While in China, fenugreek is combined with more phytomedicine in some drugs such as Qiangyang Baoshen Pills, etc. (Fig. 3D). However, according to the data from State Administration for Market Regulation, there is no available food supplement of fenugreek at present, which indicates that there is a huge market to be developed in China.

Till now, 59 clinical studies have been conducted to investigate the effect of fenugreek on human lactation, diabetes or blood glucose, sexual function, and inflammation diseases. As shown in Fig. 3E, the US and India have led the clinical studies of fenugreek since 1988, accounting for 49.2% of the total number. Meanwhile, though a long-history use in China, there is only one clinical trial reported until now. In particular, the outcomes from human investigations highlight the potential benefits of fenugreek in controlling high blood glucose and lipid levels in people with diabetes [84].

Interestingly, the west and the east have been seeking commonness among differences. Fenugreek is traditionally known as a galactagogue [85], while in China it is used to improve sexual function. Though there is no evidence show the lactation promoting effect of fenugreek in China, fenugreek supplements claiming breast milk promotion have become the best-selling products among fenugreek functional online products in China [79]. A randomized, double-blind study involved 80 healthy menstruating women with low sexual drive to evaluate the effect of fenugreek on sexual function [86]. Those women received 600 mg/day Libifem, a specialized fenugreek extract, experienced a significant increase in estradiol E2 (66%) and free testosterone (23.8%) as well as sexual desire. The reciprocal recognition of fenugreek may be attributed, at least partially, to the estrogenic activity of fenugreek. Moreover, though fenugreek is commonly recognized as a safe phytomedicine, anti-fertility, anti-implantation, and abortifacient effects were observed in animal studies, which is a warning sign for the use in pregnant women. To avoid the toxicity caused by overdose of fenugreek, the intake of fenugreek is suggested not to exceed 21 g per adult weighting 60 kg [87].

## Future perspectives

The global introduction of phytomedicine has condensed global wisdoms, experience and practices, spawning diversified applications through pharmaceutical, cosmetic, and functional food industries. Over the past decades, the growing interest of phytomedicine has refueled the scientific understanding and novel product development, while to move forward we may have to take one step backward.

Chinese medicine differs from the western in the combination of different phytomedicine for a specific clinical need. Western approach often uses one or two phytomedicine to treat symptoms, while a Chinese practitioner prefers herbal formulae where several herbs act synergistically to address the complicated and transformable conditions of patients [88]. For instance, *R. rosea* extract has been developed by many companies into various formulation of dietary supplements, while *R. crenulata* is mainly used in China in combination with other tonic herbs (Fig. 1B). However, although the compatibility of Chinese medicine has quite a long history with mature experience, some compatibility of phytomedicine in the west are still worth for the Chinese scientists or practitioners to learn from. For instance, turmeric is often used in combination with notoginseng root, Chinese angelica, safflower or white peony in China [33]. By contrast, in western countries, turmeric is now often used in combination with the black pepper based on the scientific findings, which indicate that the poor bioavailability of curcumin from turmeric can be enhanced by 20 times when combined with the piperine, an active compound in black pepper [89, 90]. Therefore, the Chinese scientists or practitioner could consider optimizing the compatibility to gain a better application with the western compatibility for reference. Likewise, the western phytomedicine system could also consider adopting the compatibility of Chinese phytomedicine. Meanwhile, processing represents another unique Chinese pharmaceutic technique to improve the use of Chinese medicine according to the theory of traditional Chinese medicine [91]. Most Chinese medicine need to be processed before their consumption, which is quite different from the phytomedicine in other western countries [92].

Furthermore, increasing number of companies are providing commercial products of phytomedicine, resulting in a booming demand of herbal materials. For those phytomedicine without large-scale artificial cultivation, like *R. rosea* and *R. crenulata*, their overall resources are on the verge of exhaustion in many main producing countries. That has led, unsurprisingly, to frequently reported adulteration, as well as low‐quality and even unsafe products [13]. As many adulterants are difficult to discriminate, there is an urgent need not only to develop artificial cultivation technologies, but also a consolidated worldwide program for quality assessment of phytomedicine [93].

From another point of view, many western traditional phytomedicine are wildly distributed but with limited medical use in China. For example, red clover (*Trifolium pratense* L.) is traditionally used as an anticancer treatment and to relieve respiratory spasm in Europe and the US. Recently, as modern scientific research reveals its potential benefit for women menopausal disorders, red clover has become a popular food supplement among western countries [94, 95]. Whereas in China, red clover is cultivated mainly as forage plant in serval provinces with little medicinal use. Therefore, in the context of the frequent global communication, we need to seize this opportunity to promote the development of phytomedicine. Meanwhile, for those western well-applied phytomedicine which do not wildly grow or is not being cultivated in China, they could be considered being introduced to China for medicinal use. A successful example is milk thistle (*Silybum marianum* L.), a common phytomedicine long been used in the treatment of liver, spleen, kidney and gallbladder disease in western countries. In recent years, milk thistle has even become one of the top-selling herbal food supplements in US. While in China, this top-selling phytomedicine has been introduced from Germany until 1972 due to its medicinal value. It is now documented in Chinese Pharmacopoeia and clinically used for treating liver diseases and China has even become one of the main cultivated sources of milk thistle worldwide [96, 97]. On the other hand, though the global introduction endows phytomedicine with more opportunities, excessive localization should also be paid attention to. A very recent example is *Lepidium meyenii* Walp., commonly known as maca or Peruvian ginseng, one of the flagship products Peru [98]. Maca has been used in Peru for at least 2000 years for its high nutritional value, while it is now widely cultivated in southwest China through an aggressive commercial promotion. Since approved by the National Health Commission as a new food resource in 2011, Chinese maca has become the main competitor of Peru [99]. Maca gained popularity in China as a sexual enhancer, while though tremendous efforts have been made in proving the sexual enhancing effect of maca thus far, supportive evidence from human studies is quite limited [100]. Excessive localization without a well-defined usage may break the long-established value chain.

The global market of phytomedicine continues to expand, while the internationalization of phytomedicine is still facing registration and policy barriers. As reported by the WHO, there are over 90 countries and regions have national polices and regulations for the marketing entry of products of phytomedicine, and the regulations of phytomedicine products are country specific [101]. China has a time-honored tradition and strong enthusiasm in using phytomedicine to keep body status and regulate body functions. The Chinese government has also issued a serial of supportive measures to promote the development of Chinese medicine, and Chinese medicine are practiced in China side-by-side with conventional medicine for healthcare. In China, Chinese medicine have been developed into thousands of Chinese patent drugs, and up to 2711 kinds of Chinese phytomedicine, extracts and Chinese patent drugs are recorded in the latest version of Chinese Pharmacopoeia. Besides, China also published an official list of phytomedicine that can be developed into functional foods. In the EU, the definition of traditional herbal medicinal products (THMP) was harmonized in the European Directive 2004/24/EC and phytomedicine with a longstanding historical use can be registered via a simplified registration pathway. The simplified pathway has provided opportunity for phytomedicine from the east to enter EU market in an expedited manner. For instance, an herbal medicinal product from China named *Diao Xinxuekang Capsules* was successfully licensed in 2012. With respect to the phytomedicine-derived food supplements, they are regulated as foods that cannot exert a pharmacological, immunological or metabolic action in the EU. Moreover, for these phytomedicine that are known or suspected to have adverse effects, such as Ephedra species and Yohimbe, the European Commission also harmonized rules to control the use. In the US, there is currently no specific regulation for phytomedicine. A product containing plant-derived ingredients that is intended to diagnose, mitigate, treat, or cure a disease is defined as botanical drug [102]. In 2004, the U.S. Food and Drug Administration (FDA) published the “Guidance for Industry: Botanical Drug Products” and a “totality-of-evidence” approach was developed. Botanical drugs are regulated in the US like other drugs, which can be sold as over the counter drugs or prescription drugs. For phytomedicine that are developed as dietary supplements, they are deemed to be foods under the Dietary Supplement Health and Education Act (DSHEA) of 1994, which are not allowed to be represented as conventional food or claimed to diagnose, treat, cure, or prevent any disease.

Nowadays, although there are many opportunities for the western phytomedicine system and the eastern phytomedicine system to communicate with each other, if a system really plans to adopt a new phytomedicine, even if this phytomedicine already has a long history of traditional application in another phytomedicine system, evidence from modern clinical studies to support it is necessary. Clearly, there are not enough clinical studies until now, especially in China. Take fenugreek for example, although with a long-used history, there is only one clinical study conducted in China so far. Beyond that, high-level clinical evidence is the key for the phytomedicine to be accepted by another system. One crucial reason why the Chinese herbal medicine is hard to break into the west is that strong clinical evidence is still lacking [103]. Therefore, more efforts should be put into conducting the clinical studies with high-level evidence for the phytomedicine.

## Conclusion

Although “East is East and West is West”, the western and eastern thinking and practices have met in the realm of phytomedicine [104]. The persistence of traditional practices of phytomedicine in both western and eastern settings is providing enormous potential for each other to learn from. As we shown in this review, the east and the west may use different species of the same genus for the same conditions or dispose different diseases by using a same herb, while the current scientific evidence to bridge the gap is still lacking. We believe the integration of the wisdom of the east and the west would generate a highly rewarding step forward to new drugs or health products.

## Data Availability

Not applicable.

## References

[1] Sorkin BC, Kuszak AJ, Bloss G, Fukagawa NK, Hoffman FA, Jafari M, Barrett B, Brown PN, Bushman FD, Casper SJ, Chilton FH, Coffey CS, Ferruzzi MG, Hopp DC, Kiely M, Lakens D, MacMillan JB, Meltzer DO, Pahor M, Paul J, Pritchett-Corning K, Quinney SK, Rehermann B, Setchell KDR, Sipes NS, Stephens JM, Taylor DL, Tiriac H, Walters MA, Xi D, Zappala G, Pauli GF (2020). Improving natural product research translation: from source to clinical trial. FASEB J.

[2] Quave CL, Pardo-de-Santayana M, Pieroni A (2012). Medical ethnobotany in Europe: from field ethnography to a more culturally sensitive evidence-based CAM?. Evid Based Complement Alternat Med.

[3] Tabajara de Oliveira Martins D, Rodrigues E, Casu L, Benitez G, Leonti M (2019). The historical development of pharmacopoeias and the inclusion of exotic herbal drugs with a focus on Europe and Brazil. J Ethnopharmacol.

[4] Booker A, Zhai L, Gkouva C, Li S, Heinrich M (2016). From traditional resource to global commodities: a comparison of rhodiola species using NMR spectroscopy-metabolomics and HPTLC. Front Pharmacol.

[5] Cui JL, Guo TT, Ren ZX, Zhang NS, Wang ML (2015). Diversity and antioxidant activity of culturable endophytic fungi from alpine plants of *Rhodiola crenulata*, *R. angusta*, and *R. sachalinensis*. PLoS ONE.

[6] Ishaque S, Shamseer L, Bukutu C, Vohra S (2012). *Rhodiola rosea* for physical and mental fatigue: a systematic review. BMC Complement Altern Med.

[7] Cunningham AB, Li HL, Luo P, Zhao WJ, Long XC, Brinckmann JA (2020). There “ain't no mountain high enough”?: The drivers, diversity and sustainability of China's Rhodiola trade. J Ethnopharmacol.

[8] Chiang HM, Chen HC, Wu CS, Wu PY, Wen KC (2015). Rhodiola plants: chemistry and biological activity. J Food Drug Anal.

[9] Kosanovic D, Tian X, Pak O, Lai YJ, Hsieh YL, Seimetz M, Weissmann N, Schermuly RT, Dahal BK (2013). Rhodiola: an ordinary plant or a promising future therapy for pulmonary hypertension? a brief review. Pulm Circ.

[10] Li T, Zhang H (2008). Identification and comparative determination of Rhodionin in traditional Tibetan medicinal plants of fourteen *Rhodiola* species by high-performance liquid chromatography-photodiode array detection and electrospray ionization-mass spectrometry. Chem Pharm Bull (Tokyo).

[11] Wang Y, Tao H, Huang H, Xiao Y, Wu X, Li M, Shen J, Xiao Z, Zhao Y, Du F, Ji H, Chen Y, Cho CH, Wang Y, Wang S, Wu X (2021). The dietary supplement *Rhodiola crenulata* extract alleviates dextran sulfate sodium-induced colitis in mice through anti-inflammation, mediating gut barrier integrity and reshaping the gut microbiome. Food Funct.

[12] Grech-Baran M, Syklowska-Baranek K, Pietrosiuk A (2015). Biotechnological approaches to enhance salidroside, rosin and its derivatives production in selected *Rhodiola* spp. in vitro cultures. Phytochem Rev.

[13] Xin T, Li X, Yao H, Lin Y, Ma X, Cheng R, Song J, Ni L, Fan C, Chen S (2015). Survey of commercial *Rhodiola* products revealed species diversity and potential safety issues. Sci Rep.

[14] Gonpo YY (1983). Si Bu Yi Dian.

[15] Dge-bśes D-D (1986). Jing Zhu Ben Cao.

[16] Tao H, Wu X, Cao J, Peng Y, Wang A, Pei J, Xiao J, Wang S, Wang Y (2019). *Rhodiola* species: a comprehensive review of traditional use, phytochemistry, pharmacology, toxicity, and clinical study. Med Res Rev.

[17] Zhang YZ, Zhu RW, Zhong DL, Zhang JQ (2018). Nunataks or massif de refuge? A phylogeographic study of *Rhodiola crenulata* (Crassulaceae) on the world's highest sky islands. BMC Evol Biol.

[18] Panossian A, Wikman G, Sarris J (2010). Rosenroot (*Rhodiola rosea*): traditional use, chemical composition, pharmacology and clinical efficacy. Phytomedicine.

[19] Kosakowska O, Baczek K, Przybyl JL, Pioro-Jabrucka E, Czupa W, Synowiec A, Gniewosz M, Costa R, Mondello L, Weglarz Z (2018). Antioxidant and antibacterial activity of roseroot (*Rhodiola rosea* L.) dry extracts. Molecules.

[20] Brown RP, Gerbarg PL, Ramazanov Z (2002). *Rhodiola rosea*: a phytomedicinal overview. HerbalGram.

[21] Li Y, Pham V, Bui M, Song L, Wu C, Walia A, Uchio E, Smith-Liu F, Zi X (2017). *Rhodiola rosea* L.: an herb with anti-stress, anti-aging, and immunostimulating properties for cancer chemoprevention. Curr Pharmacol Rep.

[22] Recio MC, Giner RM, Manez S (2016). Immunmodulatory and antiproliferative properties of *Rhodiola* Species. Planta Med.

[23] Galambosi B (2005). *Rhodiola rosea* L., from wild collection to field production. Med Plant Conserv.

[24] Ruhsam M, Hollingsworth PM (2018). Authentication of *Eleutherococcus* and *Rhodiola* herbal supplement products in the United Kingdom. J Pharm Biomed Anal.

[25] Booker A, Jalil B, Frommenwiler D, Reich E, Zhai L, Kulic Z, Heinrich M (2016). The authenticity and quality of *Rhodiola rosea* products. Phytomedicine.

[26] Lei Y, Gao H, Tsering T, Shi S, Zhong Y (2006). Determination of genetic variation in *Rhodiola crenulata* from the Hengduan Mountains Region, China using inter-simple sequence repeats. Genet Mol Biol.

[27] Galambosi B (2006). Demand and availability of *Rhodiola rosea* L. raw material. Medicinal and aromatic plants.

[28] Li FYY, Li H, Zhan Z, Kang L, Li M (2015). Infrared-assisted extraction of salidroside from the root of *Rhodiola crenulata* with a novel ionic liquid that dissolves cellulose. RSC Adv.

[29] Lee SY, Shi LS, Chu H, Li MH, Ho CW, Lai FY, Huang CY, Chang TC (2013). *Rhodiola crenulata* and its bioactive components, salidroside and tyrosol, reverse the hypoxia-induced reduction of plasma-membrane-associated Na, K-ATPase expression via inhibition of ROS-AMPK-PKC xi pathway. Evid Based Complement Alternat Med.

[30] Pu WL, Zhang MY, Bai RY, Sun LK, Li WH, Yu YL, Zhang Y, Song L, Wang ZX, Peng YF, Shi H, Zhou K, Li TX (2020). Anti-inflammatory effects of *Rhodiola rosea* L.: a review. Biomed Pharmacother.

[31] Zhang W, Huai Y, Miao Z, Chen C, Shahen M, Rahman SU, Alagawany M, El-Hack MEA, Zhao H, Qian A (2019). Systems pharmacology approach to investigate the molecular mechanisms of herb *Rhodiola rosea* L. radix. Drug Dev Ind Pharm.

[32] Darbinyan V, Kteyan A, Panossian A, Gabrielian E, Wikman G, Wagner H (2000). *Rhodiola rosea* in stress induced fatigue–a double blind cross-over study of a standardized extract SHR-5 with a repeated low-dose regimen on the mental performance of healthy physicians during night duty. Phytomedicine.

[33] Commission (2015). Chinese pharmacopoeia.

[34] Abidov M, Crendal F, Grachev S, Seifulla R, Ziegenfuss T (2003). Effect of extracts from *Rhodiola rosea* and *Rhodiola crenulata* (Crassulaceae) roots on ATP content in mitochondria of skeletal muscles. Bull Exp Biol Med.

[35] Kwon YI, Jang HD, Shetty K (2006). Evaluation of *Rhodiola crenulata* and *Rhodiola rosea* for management of type II diabetes and hypertension. Asia Pac J Clin Nutr.

[36] Teng H, Chen L (2017). alpha-Glucosidase and alpha-amylase inhibitors from seed oil: a review of liposoluble substance to treat diabetes. Crit Rev Food Sci Nutr.

[37] Yunuskhodjaev A, Iskandarova S, Kurmukov A, Saidov S (2014). Study of adaptogenic properties and chronic toxicity of extract of *Rhodiola heterodonta*. Eur J Nat Hist.

[38] Gupta V, Saggu S, Tulsawani RK, Sawhney RC, Kumar R (2008). A dose dependent adaptogenic and safety evaluation of *Rhodiola imbricata* Edgew, a high altitude rhizome. Food Chem Toxicol.

[39] Diyong C (1998). Study on the long term toxicology of Tibet Rhodiola sacera. J North Sichuan Med Coll.

[40] Montiel-Ruiz RM, Roa-Coria JE, Patino-Camacho SI, Flores-Murrieta FJ, Deciga-Campos M (2012). Neuropharmacological and toxicity evaluations of ethanol extract from *Rhodiola rosea*. Drug Dev Res.

[41] Cropley M, Banks AP, Boyle J (2015). The effects of *Rhodiola rosea* L. extract on anxiety, stress, cognition and other mood symptoms. Phytother Res.

[42] Punja S, Shamseer L, Olson K, Vohra S (2014). Rhodiola rosea for mental and physical fatigue in nursing students: a randomized controlled trial. PLoS ONE.

[43] Hu K, Wang Z, Li Y, Yu X, Wang J (1998). Clinical report of 100 cases of asthenia-syndrome treated with radix Rhodiola capsule. Journal of Chengdu University of Traditional Chinese Medicine..

[44] Gao XQ, Ohlander M, Jeppsson N, Bjork L, Trajkovski V (2000). Changes in antioxidant effects and their relationship to phytonutrients in fruits of sea buckthorn (*Hippophae rhamnoides* L.) during maturation. J Agric Food Chem.

[45] Ruan CJ, Rumpunen K, Nybom H (2013). Advances in improvement of quality and resistance in a multipurpose crop: sea buckthorn. Crit Rev Biotechnol.

[46] Ma X, Laaksonen O, Zheng J, Yang W, Trepanier M, Kallio H, Yang B (2016). Flavonol glycosides in berries of two major subspecies of sea buckthorn (*Hippophae rhamnoides* L) and influence of growth sites. Food Chem.

[47] Letchamo W, Ozturk M, Altay V, Musayev M, Mamedov NA, Hakeem KR (2018). An alternative potential natural genetic resource: sea buckthorn [*Elaeagnus rhamnoides* (syn: *Hippophae rhamnoides*)]. Global perspectives on underutilized crops.

[48] Sayegh M, Miglio C, Ray S (2014). Potential cardiovascular implications of Sea Buckthorn berry consumption in humans. Int J Food Sci Nutr.

[49] Guliyev VB, Gul M, Yildirim A (2004). *Hippophae rhamnoides* L.: chromatographic methods to determine chemical composition, use in traditional medicine and pharmacological effects. J Chromatogr B Analyt Technol Biomed Life Sci.

[50] Guo R, Guo X, Li T, Fu X, Liu RH (2017). Comparative assessment of phytochemical profiles, antioxidant and antiproliferative activities of Sea buckthorn (*Hippophae rhamnoides* L.) berries. Food Chem.

[51] Sola Marsinach M, Cuenca AP (2019). The impact of sea buckthorn oil fatty acids on human health. Lipids Health Dis.

[52] He C, Zhang G, Zhang J, Zeng Y, Liu J (2017). Integrated analysis of multiomic data reveals the role of the antioxidant network in the quality of sea buckthorn berry. FASEB J.

[53] Zeb A (2004). Important therapeutic uses of sea buckthorn (Hippophae): a review. J Biol Sci.

[54] Wani TA, Wani S, Ahmad M, Ahmad M, Gani A, Masoodi F (2016). Bioactive profile, health benefits and safety evaluation of sea buckthorn (*Hippophae rhamnoides* L.): a review. Cogent Food Agric.

[55] Zhao P, Wang SL, Liang C, Wang YW, Wen PJ, Wang F, Qin GQ (2017). Acute and subchronic toxicity studies of seabuckthorn (*Hippophae rhamnoides* L.) oil in rodents. Regul Toxicol Pharmacol.

[56] Upadhyay NK, Kumar R, Mandotra SK, Meena RN, Siddiqui MS, Sawhney RC, Gupta A (2009). Safety and healing efficacy of Sea buckthorn (*Hippophae rhamnoides* L.) seed oil on burn wounds in rats. Food Chem Toxicol.

[57] Larmo P, Jarvinen R, Laihia J, Loyttyniemi E, Maavirta L, Yang B, Kallio H, Sandberg-Lall M (2019). Effects of a sea buckthorn oil spray emulsion on dry eye. Cont Lens Anterior Eye.

[58] Vashishtha V, Barhwal K, Kumar A, Hota SK, Chaurasia OP, Kumar B (2017). Effect of seabuckthorn seed oil in reducing cardiovascular risk factors: a longitudinal controlled trial on hypertensive subjects. Clin Nutr.

[59] Huang NK, Matthan NR, Galluccio JM, Shi P, Lichtenstein AH, Mozaffarian D (2020). Supplementation with seabuckthorn oil augmented in 16:1n–7t increases serum trans-palmitoleic acid in metabolically healthy adults: a randomized crossover dose-escalation study. J Nutr.

[60] Larmo PS, Yang B, Hyssala J, Kallio HP, Erkkola R (2014). Effects of sea buckthorn oil intake on vaginal atrophy in postmenopausal women: a randomized, double-blind, placebo-controlled study. Maturitas.

[61] Suryakumar G, Gupta A (2011). Medicinal and therapeutic potential of Sea buckthorn (*Hippophae rhamnoides* L.). J Ethnopharmacol.

[62] Olas B (2016). Sea buckthorn as a source of important bioactive compounds in cardiovascular diseases. Food Chem Toxicol.

[63] Kumar R, Kumar GP, Chaurasia O, Singh SB (2011). Phytochemical and pharmacological profile of seabuckthorn oil: a review. Res J Med Plant.

[64] Olas B (2018). The beneficial health aspects of sea buckthorn (*Elaeagnus rhamnoides* (L.) A.Nelson) oil. J Ethnopharmacol.

[65] Li TS (2002). Product development of sea buckthorn. Trends in new crops and new uses.

[66] Bal LM, Meda V, Naik SN, Satya S (2011). Sea buckthorn berries: a potential source of valuable nutrients for nutraceuticals and cosmoceuticals. Food Res Int.

[67] Geetha S, Ram MS, Singh V, Ilavazhagan G, Sawhney RC (2002). Anti-oxidant and immunomodulatory properties of seabuckthorn (*Hippophae rhamnoides*)—an in vitro study. J Ethnopharmacol.

[68] Geetha S, Jayamurthy P, Pal K, Pandey S, Kumar R, Sawhney RC (2008). Hepatoprotective effects of sea buckthorn (*Hippophae rhamnoides* L.) against carbon tetrachloride induced liver injury in rats. J Sci Food Agric.

[69] Hsu YW, Tsai CF, Chen WK, Lu FJ (2009). Protective effects of seabuckthorn (*Hippophae rhamnoides* L.) seed oil against carbon tetrachloride-induced hepatotoxicity in mice. Food Chem Toxicol.

[70] Jain M, Ganju L, Katiyal A, Padwad Y, Mishra KP, Chanda S, Karan D, Yogendra KMS, Sawhney RC (2008). Effect of *Hippophae rhamnoides* leaf extract against Dengue virus infection in human blood-derived macrophages. Phytomedicine.

[71] Xing JF, Yang BR, Dong YL, Wang BW, Wang JX, Kallio HP (2002). Effects of sea buckthorn (*Hippophae rhamnoides* L.) seed and pulp oils on experimental models of gastric ulcer in rats. Fitoterapia.

[72] Basu M, Prasad R, Jayamurthy P, Pal K, Arumughan C, Sawhney RC (2007). Anti-atherogenic effects of seabuckthorn (*Hippophaea rhamnoides*) seed oil. Phytomedicine.

[73] Krejcarova J, Strakova E, Suchy P, Herzig I, Karaskova K (2015). Sea buckthorn (*Hippophae rhamnoides* L.) as a potential source of nutraceutics and its therapeutic possibilities—a review. Acta Vet Brno.

[74] Beveridge T, Li TS, Oomah BD, Smith A (1999). Sea buckthorn products: manufacture and composition. J Agric Food Chem.

[75] Panossian A, Wagner H, Panossian A (2013). From traditional to evidence-based use of *Hippophae rhamnoides* L.: chemical composition, experimental, and clinical pharmacology of sea buckthorn berries and leaves extracts. Evidence and rational based research on Chinese drugs.

[76] Larmo PS, Jarvinen RL, Setala NL, Yang B, Viitanen MH, Engblom JR, Tahvonen RL, Kallio HP (2010). Oral sea buckthorn oil attenuates tear film osmolarity and symptoms in individuals with dry eye. J Nutr.

[77] Jarvinen RL, Larmo PS, Setala NL, Yang B, Engblom JR, Viitanen MH, Kallio HP (2011). Effects of oral sea buckthorn oil on tear film fatty acids in individuals with dry eye. Cornea.

[78] Acharya SN, Thomas JE, Basu SK (2006). Fenugreek: an “old world” crop for the “new world”. Biodiversity.

[79] Yao D, Zhang B, Zhu J, Zhang Q, Hu Y, Wang S, Wang Y, Cao H, Xiao J (2019). Advances on application of fenugreek seeds as functional foods: pharmacology, clinical application, products, patents and market. Crit Rev Food Sci Nutr.

[80] Gong J, Fang K, Dong H, Wang D, Hu M, Lu F (2016). Effect of fenugreek on hyperglycaemia and hyperlipidemia in diabetes and prediabetes: a meta-analysis. J Ethnopharmacol.

[81] Ahmad A, Alghamdi SS, Mahmood K, Afzal M (2016). Fenugreek a multipurpose crop: potentialities and improvements. Saudi J Biol Sci.

[82] Basch E, Ulbricht C, Kuo G, Szapary P, Smith M (2003). Therapeutic applications of fenugreek. Altern Med Rev.

[83] Korman SH, Cohen E, Preminger A (2001). Pseudo-maple syrup urine disease due to maternal prenatal ingestion of fenugreek. J Paediatr Child Health.

[84] Najdi RA, Hagras MM, Kamel FO, Magadmi RM (2019). A randomized controlled clinical trial evaluating the effect of *Trigonella foenum-graecum* (fenugreek) versus glibenclamide in patients with diabetes. Afr Health Sci.

[85] Mortel M, Mehta SD (2013). Systematic review of the efficacy of herbal galactogogues. J Hum Lact.

[86] Rao A, Steels E, Beccaria G, Inder WJ, Vitetta L (2015). Influence of a specialized *Trigonella foenum-graecum* SEED EXTRACT (libifem), on testosterone, estradiol and sexual function in healthy menstruating women, a randomised placebo controlled study. Phytother Res.

[87] Ouzir M, El Bairi K, Amzazi S (2016). Toxicological properties of fenugreek (*Trigonella foenum graecum*). Food Chem Toxicol.

[88] Wang S, Hu Y, Tan W, Wu X, Chen R, Cao J, Chen M, Wang Y (2012). Compatibility art of traditional Chinese medicine: from the perspective of herb pairs. J Ethnopharmacol.

[89] Hewlings SJ, Kalman DS (2017). Curcumin: a review of its' effects on human health. Foods.

[90] Gupta SC, Kismali G, Aggarwal BB (2013). Curcumin, a component of turmeric: from farm to pharmacy. BioFactors.

[91] Wu X, Wang S, Lu J, Jing Y, Li M, Cao J, Bian B, Hu C (2018). Seeing the unseen of Chinese herbal medicine processing (Paozhi): advances in new perspectives. Chin Med.

[92] Heinrich M, Scotti F, Booker A, Fitzgerald M, Kum KY, Lobel K (2019). Unblocking high-value botanical value chains: is there a role for blockchain systems?. Front Pharmacol.

[93] Marchev AS, Koycheva IK, Aneva IY, Georgiev MI (2020). Authenticity and quality evaluation of different *Rhodiola* species and commercial products based on NMR-spectroscopy and HPLC. Phytochem Anal.

[94] Booth NL, Piersen CE, Banuvar S, Geller SE, Shulman LP, Farnsworth NR (2006). Clinical studies of red clover (*Trifolium pratense*) dietary supplements in menopause: a literature review. Menopause.

[95] Mueller M, Jungbauer A (2008). Red clover extract: a putative source for simultaneous treatment of menopausal disorders and the metabolic syndrome. Menopause.

[96] Abenavoli L, Capasso R, Milic N, Capasso F (2010). Milk thistle in liver diseases: past, present, future. Phytother Res.

[97] Abenavoli L, Izzo AA, Milic N, Cicala C, Santini A, Capasso R (2018). Milk thistle (*Silybum marianum*): a concise overview on its chemistry, pharmacological, and nutraceutical uses in liver diseases. Phytother Res.

[98] da Silva Leitao Peres N, Cabrera Parra Bortoluzzi L, Medeiros Marques LL, Formigoni M, Fuchs RHB, Droval AA, Reitz Cardoso FA (2020). Medicinal effects of Peruvian maca (*Lepidium meyenii*): a review. Food Funct.

[99] Heinrich M, Hesketh A (2019). 25 years after the `Rio Convention'–lessons learned in the context of sustainable development and protecting indigenous and local knowledge. Phytomedicine.

[100] Shin BC, Lee MS, Yang EJ, Lim HS, Ernst E (2010). Maca (*L. meyenii*) for improving sexual function: a systematic review. BMC Complement Altern Med.

[101] Organization WH. Legal status of traditional medicine and complementary (No. WHO/EDM/TRM/2001.2)*. *World Health Organization; 2001. https://apps.who.int/iris/handle/10665/42452.

[102] Li JT, Zhu JF, Hu H, Harnett JE, Lei CI, Chau KY, Chan G, Ung COL (2018). Internationalization of Traditional/Complementary Medicine products: market entry as medicine. Chin Med.

[103] Fung FY, Linn YC (2015). Developing traditional chinese medicine in the era of evidence-based medicine: current evidences and challenges. Evid Based Complement Alternat Med..

[104] Fan T-P, Briggs J, Liu L, Lu A, Greef JVD, Xu A (2014). The art and science of traditional medicine part 1: TCM today-a case for integration. Science.

