# Correction to: West meets east: open up a dialogue on phytomedicine

**DOI:** 10.1186/s13020-021-00490-7

**Published:** 2021-08-17

**Authors:** Xiuzhu Li, Weijie Chen, Jesus Simal-Gandara, Milen I. Georgiev, Hongyi Li, Hao Hu, Xu Wu, Thomas Eferth, Shengpeng Wang

**Affiliations:** 1 Institute of Chinese Medical Sciences, State Key Laboratory of Quality Research in Chinese Medicine, University of Macau, Taipa, Macao SAR China; 2 Nutrition and Bromatology Group, Department of Analytical Chemistry and Food Science, Faculty of Food Science and Technology, University of VigoOurense Campus, 32004 Ourense, Spain; 3 Laboratory of Metabolomics, The Stephan Angelof Institute of Microbiology, Bulgarian Academy of Sciences, Plovdiv, Bulgaria; 4 Laboratory of Molecular Pharmacology, Department of Pharmacology, School of Pharmacy, Southwest Medical University, Luzhou, Sichuan China; 5 Institute of Pharmaceutical and Biomedical Sciences, Department of Pharmaceutical Biology, Johannes Gutenberg University, Mainz, Germany

## Correction to: Chin Med (2021) 16:57 10.1186/s13020-021-00467-6

Following the publication of the original article [1], the authors identified an error in Fig. 1. The bubbles in Fig. 1D are missing

The correct figure (Fig. 1) has been included in this correction, and the original article has been corrected.

**Fig. 1:**
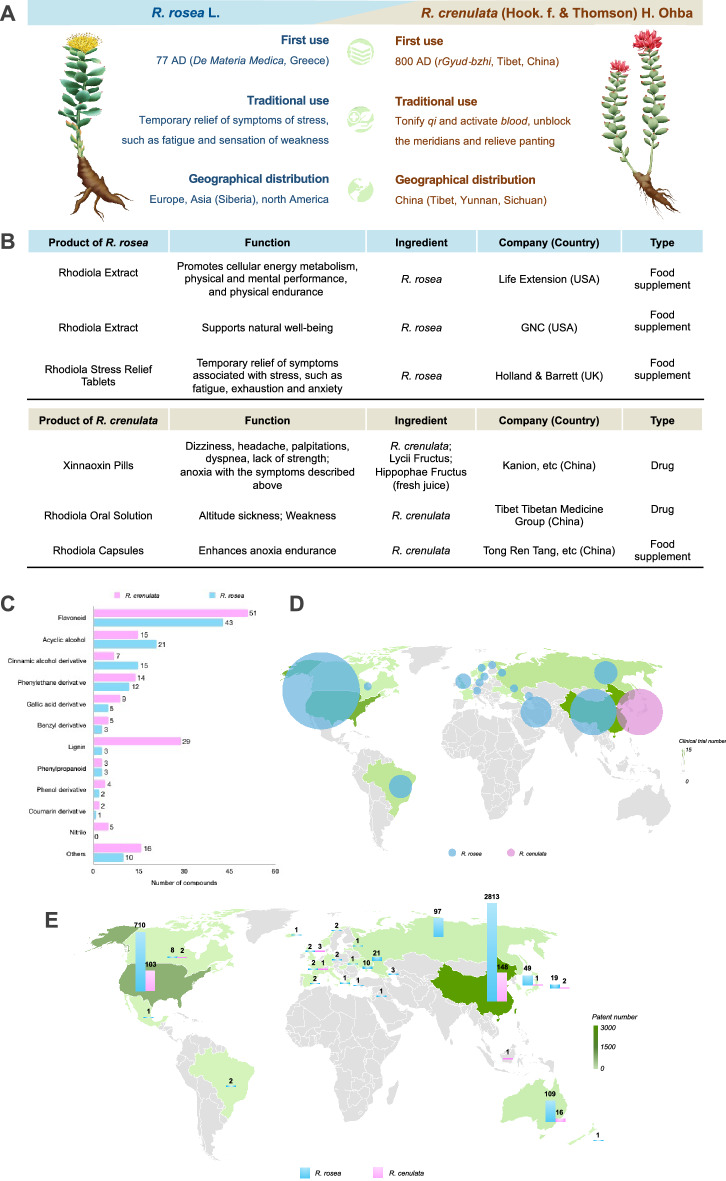
Comparison of history, products, active constituents, clinical trials and patens of two *Rhodiola* species. **A** Basic information of *R. crenulata* and *R. rosea*. **B** Representative products developed based on *R. crenulata* and *R. rosea*. **C** Number of main active constituents contained in *R. crenulata* and *R. rosea*. **D** Advanced in clinical trials of *R. crenulata* and *R. rosea*. Data collected from PubMed (searching term of “*Rhodiola*” plus flter of “Clinical trial”, language limited to English) and ClinicalTrials.gov (searching term of “Rhodiola”) as of 15 June 2021. **E** Patent application of *R. crenulata* and *R. rosea*. Data collected from the Lens (https://www.lens.org, Searching term: *Rhodiola*) as of 15 June 2021

## References

[1] Li X, Chen W, Simal-Gandara J, Georgiev MI, Li H, Hu H, Wu X, Efferth T, Wang S (2021). West meets east: open up a dialogue on phytomedicine. Chin Med.

